# The role of Ca^2+^ signaling in Parkinson's disease

**DOI:** 10.1242/dmm.028738

**Published:** 2017-05-01

**Authors:** Sofia V. Zaichick, Kaitlyn M. McGrath, Gabriela Caraveo

**Affiliations:** Ken and Ruth Davee Department of Neurology, Feinberg School of Medicine, Northwestern University, Chicago, IL 60611, USA

**Keywords:** Calcium, α-synuclein, Parkinson's disease

## Abstract

Across all kingdoms in the tree of life, calcium (Ca^2+^) is an essential element used by cells to respond and adapt to constantly changing environments. In multicellular organisms, it plays fundamental roles during fertilization, development and adulthood. The inability of cells to regulate Ca^2+^ can lead to pathological conditions that ultimately culminate in cell death. One such pathological condition is manifested in Parkinson's disease, the second most common neurological disorder in humans, which is characterized by the aggregation of the protein, α-synuclein. This Review discusses current evidence that implicates Ca^2+^ in the pathogenesis of Parkinson's disease. Understanding the mechanisms by which Ca^2+^ signaling contributes to the progression of this disease will be crucial for the development of effective therapies to combat this devastating neurological condition.

## Abstract (precis)

**Summary:** This Review article discusses the important roles that calcium plays in the pathogenesis of Parkinson's disease.

## Introduction

Parkinson's disease (PD) is the second most common, multifactorial, progressive neurodegenerative disorder in humans after Alzheimer's disease, affecting 6.3 million people worldwide (Marras and Tanner, 2004). Characterized by the aggregation of a small lipid-binding protein, α-synuclein, PD belongs to a larger group of neurodegenerative diseases, collectively known as synucleinopathies. This group includes dementia with Lewy bodies (DLB), neurodegeneration with brain iron accumulation and multiple system atrophy (MSA) (Martí et al., 2003; Teive et al., 2004). Although the common theme amongst these synucleinopathies is α-synuclein aggregation into structures called Lewy bodies, the pathological distinction between each disorder lies primarily in the cell type affected. In MSA and DLB, Lewy bodies are primarily found in oligodendrocytes and cortical neurons, respectively. In PD, Lewy bodies are detected primarily in dopaminergic (DA) neurons in a brain region called the substantia nigra pars compacta (SNc). Although it is true that the motor symptoms observed in PD, such as resting tremor, bradikinesia and postural rigidity, can be ascribed to the loss of DA neurons in the SNc, it is now very clear that there are many other brain regions with Lewy body pathology. In fact, many of these regions correspond to the non-motor symptoms that often precede the motor symptoms of PD, such as apathy, pain, sexual difficulties, constipation and sleep disorders, among others (Braak et al., 2004; Chaudhuri et al., 2006; Lees et al., 2009). The pathological overlap between different synucleinopathies suggests that these diseases might belong on a spectrum of the same disorder. Therefore, it is important to understand the consequences of α-synuclein aggregation in different cell types to fully understand the scope of PD pathology.

Over the past 10 years, an explosion of research has identified over 30 genetic loci and genes responsible for PD, and the list is still growing (Table 1) (Chen et al., 2013; Ghanbari et al., 2016; Höglinger et al., 2011; Kumar et al., 2011; Lin and Farrer, 2014; Martin et al., 2011; Nalls et al., 2014; Shulman et al., 2011; Wissemann et al., 2013). Although genetic cases represent only 10% of PD, genome-wide association (GWA) studies are increasingly being used to elucidate novel risk loci for PD. These studies provide new insights into the complex interplay between genetics, epigenetics and environmental factors that contribute to PD pathology. Whether the cause of PD is genetic, environmental and/or sporadic, α-synuclein aggregation is a key pathological hallmark of the disease. Point mutations, duplication and triplication of the α-synuclein locus are known to cause the early onset of PD (Polymeropoulos et al., 1997; Simón-Sánchez et al., 2009; Singleton et al., 2003). Moreover, GWA studies have revealed that the α-synuclein gene (*SNCA*) is a major risk factor that is linked to sporadic PD (Simón-Sánchez et al., 2009).

**Table 1.**
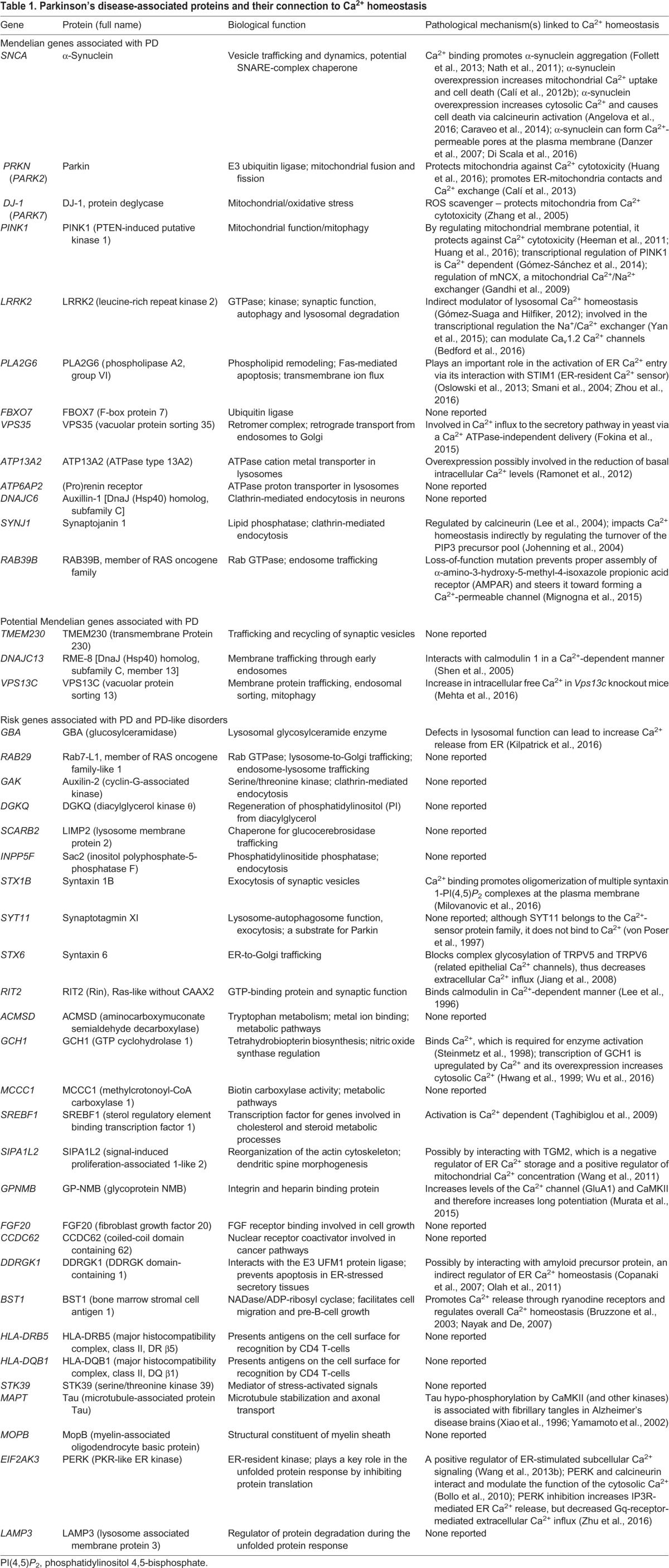
**Parkinson's disease-associated proteins and their connection to Ca^2+^ homeostasis**

An emerging, key pathological feature caused by α-synuclein aggregation is the disruption of calcium (Ca^2+^) homeostasis (Caraveo et al., 2014; Goldberg et al., 2012; Guzman et al., 2010; Hurley et al., 2013; Surmeier et al., 2010, 2016). Ca^2+^ is a universal and versatile second messenger that is present in all living organisms. Unlike Na^+^ and K^+^, which have ∼10- to 30-fold differences in ion concentration across the plasma membrane, Ca^2+^ ions have a 20,000-fold lower concentration in the cytoplasm compared to in the extracellular space (Surmeier and Schumacker, 2013). These gradients allow cells to use Ca^2+^ as a potent intracellular signal to respond and adapt to fast-changing extracellular and intracellular environments. By controlling the amplitude and frequency of Ca^2+^ dynamics, cells can temporarily or permanently change a wide variety of physiological functions by activating and/or inhibiting Ca^2+^-dependent signal transduction pathways (Berridge, 2005; Berridge et al., 2003, 2000; Bootman, 2012; Burgoyne, 2007; Carafoli, 2002; Clapham, 2007; Petersen et al., 2005; Rizzuto and Pozzan, 2006). In stimulated neurons, cytoplasmic Ca^2+^ can range from 100 nM up to 1-10 µM in selected microdomains, depending on the cell type. The polarized nature of neurons allows them to regulate specific processes that are generally not sensitive to bulk concentrations of Ca^2+^, such as neuronal development and synaptic plasticity (Augustine et al., 2003; Bootman et al., 2001; Carrasco and Hidalgo, 2006; Muller et al., 2005; Parekh, 2008). Because Ca^2+^ signaling affects all aspects of neuronal cell biology, cells must tightly regulate Ca^2+^ levels to avoid uncontrolled responses that could otherwise lead to pathological conditions and cell death (Rozkalne et al., 2011; White et al., 2000).

In this Review, we discuss the current evidence that implicates defective Ca^2+^ homeostasis in the pathogenesis of PD. Elucidating the role of α-synuclein, and of other PD-associated proteins, in Ca^2+^ homeostasis could provide new opportunities for developing novel therapeutics to treat synucleinopathies.

**Box 1. Ca^2+^ signaling and other neurodegenerative diseases**

Defects in Ca^2+^ homeostasis might also play a causal role in neurodegenerative disorders other than Parkinson's disease, such as Alzheimer’s disease (AD). Oligomeric forms of the amyloid β (Aβ)-peptide, the major component of amyloid plaques (a pathological hallmark of AD), can create pores at the plasma membrane and trigger Ca^2+^-induced toxicity (Arispe et al., 1993), similar to α-synuclein oligomers. Presenilins are a family of related multi-pass ER transmembrane proteins that constitute the catalytic subunits of the γ-secretase intramembrane protease complex. Presenilins can modify lysosomal and ER Ca^2+^ channels (Kayala et al., 2012; Nelson et al., 2011) and have been implicated in familial forms of AD (Tolia and De Strooper, 2009). Mutations in the presenilins cause severe defects in ER Ca^2+^ homeostasis through a combination of mechanisms that involve an increase in SOCE, expression of RyR and IP3R, and inhibition of Ca^2+^ leakage from the ER, leading to ER Ca^2+^ overload and, consequently, to cell death (Bezprozvanny, 2009). Decreased expression of Ca^2+^-binding buffer proteins, such as calbindin, in the hippocampus has also been directly correlated with cognitive decline in the mouse AD model (Palop et al., 2003), reminiscent of the protective role of Ca^2+^ buffering in PD. The inhibition of calcineurin by FK506 reportedly restores memory, alters behavior and increases survival in mouse models of AD (Dineley et al., 2007; Mukherjee et al., 2010; Reese et al., 2008). Finally, the Ca^2+^ homeostasis modulator, CALHM1, is also reported to be a risk gene for AD (Dreses-Werringloer et al., 2008).

Amyotrophic lateral sclerosis (ALS) is characterized by selective degeneration of motor neurons. The most compelling evidence linking Ca^2+^ defects with cell death in ALS is excitotoxicity caused by glial cells (Sasabe et al., 2007). Huntington's disease is a genetic disorder characterized by an increased number (over 40) of glutamine amino acids at the N-terminus of the huntingtin protein (Htt), which affect the medium spiny neurons. Expanded Htt binds to IP3R, which increases its sensitivity to IP3, thereby stimulating Ca^2+^ efflux from the ER (Chan et al., 2003; Tang et al., 2003).

## PD and Ca^2+^ signaling at the plasma membrane

**Fig. 1.**
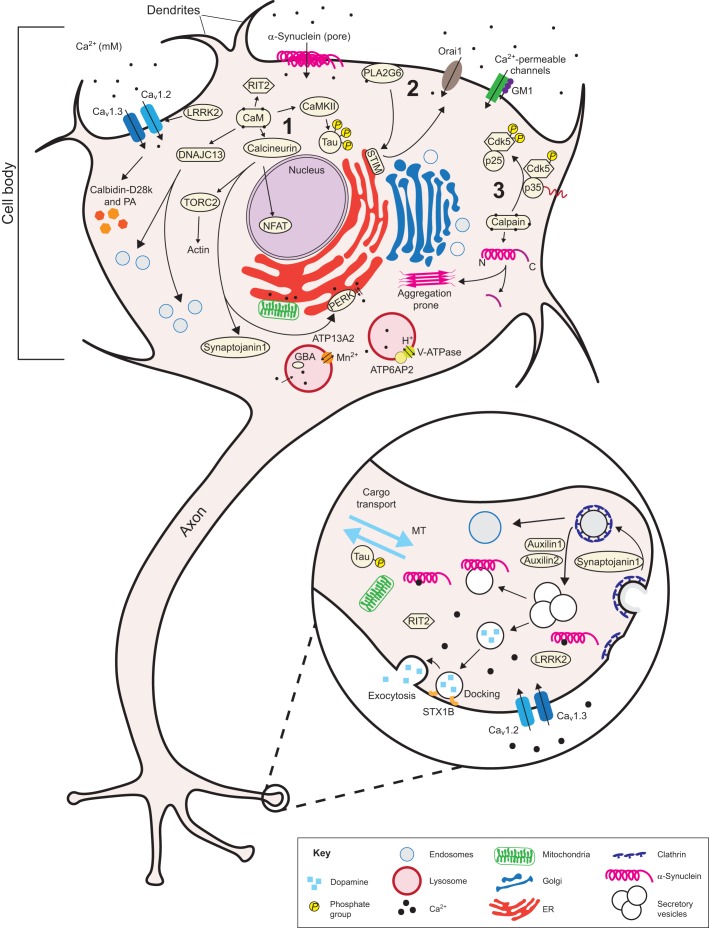
**Ca^2+^ signaling and homeostasis in a dopaminergic neuron.** A schematic of a dopamine (DA) neuron, illustrating several Ca^2+^-related proteins and pathways affected in Parkinson's disease (PD). The proteins shown directly or indirectly participate in Ca^2+^ homeostasis. Ca_v_1.2, Ca_v_1.3, Orai1, Ca^2+^-permeable channels (T-channels, NCX, TRPC5, PMCA) regulated by GM1, and α-synuclein Ca^2+^-permeable pores allow Ca^2+^ to enter the cell. Calbidin-D28k and parvalbumin (PA) are protective due to their capacity to buffer cytosolic Ca^2+^. Increases in cytosolic Ca^2+^ activate diverse pathways involved in PD, including: (1) calmodulin (CaM) and calcineurin to modify their respective downstream targets NFAT, TORC2 and synaptojanin1; (2) PLA2G6 (through SOCE); and (3) calpains. Increases in cytosolic Ca^2+^ also activate the lysosomal ion channels ATP13A2 and ATP6AP2. Lower right: A magnified pre-synaptic axonal terminal illustrates the role of RIT2, STX1B (syntaxin 1B), α-synuclein and synaptojanin1 in vesicle recycling. The role of LRRK2 in pre-synaptic vesicle recycling is not fully known. Hyperphosphorylation of Tau driven by CaMKII activation interferes with proper microtubule (MT) axonal transport. Abbreviations: ATP13A2, probable cation-transporting ATPase 13A2; ATP6AP2 (prorenin receptor), ATPase H^+^-transporting lysosomal accessory protein 2; CaMKII, calmodulin kinase II; Ca_v_1.2 and Ca_v_1.3, subunits of voltage-dependent L-type Ca^2+^ channels; Cdk5, cyclin-dependent kinase 5; DNAJC13, DnaJ homolog subfamily C member 13; ER, endoplasmic reticulum; GBA, glucocerebrosidase; GM1, monosialotetrahexosylganglioside; LRRK2, leucine-rich repeat kinase 2; NCX, Na^+^/Ca^2+^ exchanger; NFAT, nuclear factor of activated T cells; Orai1, Ca^2+^ release-activated Ca^2+^ channel protein 1; p35, cyclin-dependent kinase 5 activator encoded by *CDK5R1*; p25, a calpain cleavage product of p35; PERK, protein kinase RNA-like endoplasmic reticulum kinase; PLA2G6, phospholipase A2G6; PMCA, plasma membrane Ca^2+^ ATPase; RIT2, Ras-like without CAAX2; SOCE, store operated Ca^2+^ entry; STIM, stromal interaction molecule; TORC2, transducer of regulated CREB protein 2; TRPC5, short transient receptor potential channel 5.

In neurons, the movement of Ca^2+^ can occur across the plasma membrane in response to electrical activity and/or through agonists. The electrical activity of neurons and other excitable cells relies on several different types of voltage- and ligand-gated ion channels that are permeable to inorganic ions, such as Na^+^, K^+^, Cl^−^ and Ca^2+^. L-type (also known as Ca_v_1 family) voltage-gated Ca^2+^ channels, Ca_v_1.2 and Ca_v_1.3, have been implicated in PD (Calí et al., 2014; Hurley and Dexter, 2012; Ortner and Striessnig, 2016; Schapira, 2013; Surmeier et al., 2016; Zamponi, 2016). Although Ca_v_1.2 is prevalent in juvenile SNc DA neurons, in aging SNc DA neurons, Ca_v_1.3 is preferentially used for Ca^2+^ influx and support of rhythmic pace-making activity (Fig. 1) (Bean, 2007; Chan et al., 2007; Dragicevic et al., 2014; Drion et al., 2011; Goldberg et al., 2012; Khaliq and Bean, 2010; Puopolo et al., 2007; Surmeier et al., 2012; Wilson and Callaway, 2000). Such pace-making is essential for maintaining basal dopamine levels in the striatum (Surmeier and Schumacker, 2013). Unlike Ca_v_1.2, the Ca_v_1.3 operating range does not allow the Ca_v_1.3 channels to close fully during pace-making, which contributes to elevated intracellular Ca^2+^ levels (Puopolo et al., 2007; Wilson and Callaway, 2000). In adult mice, SNc DA neurons have an increased reliance on Ca_v_1.3 channels, as well as a decreased ability to deal with high Ca^2+^ levels (Chan et al., 2007; Hurley et al., 2013). Interestingly, the expression of Ca_v_1.3 is increased in the SNc DA neurons of deceased PD patients (Hurley et al., 2013). To test the importance of L-type channels in PD-like pathology, mice, midbrain slices or cultured neurons from mice were pretreated with Isradipine, an L-type Ca^2+^ channel blocker, and then exposed to α-synuclein pre-formed fibrils (PFF), or to the toxic effects of environmental factors known to cause PD by interfering with the mitochondrial complex I, namely rotenone or 1-methyl-4-phenyl-1,2,3,6 tetrahydropyridine (MPTP) (Brown et al., 2006; Chan et al., 2007; Dryanovski et al., 2013; Goldman, 2014; Ilijic et al., 2011; Van Maele-Fabry et al., 2012). In these experiments, Isradipine confers strong protection in SNc DA neurons, indicating that Ca^2+^ flux through L-type channels is an important contributor to neuronal cell death. The importance of this finding is also supported by the fact that the neighboring ventral tegmental midbrain DA neurons, which do not express the Ca_v_1.3 channels, are less susceptible to cell death in PD (Hurley et al., 2013; Mouatt-Prigent et al., 1994; Neuhoff et al., 2002).

In the clinic, Isradipine and other L-type channel blockers have been widely used as anti-hypertensives to treat high blood pressure and other cardiovascular conditions. The proposed role of Ca^2+^ channels in neurodegeneration opens up the possibility of repurposing these drugs to treat PD. Several epidemiological studies suggest that there is indeed a reduced risk of developing PD in patients with long-term use of Isradipine (Becker et al., 2008; Lee et al., 2014; Pasternak et al., 2012; Ritz et al., 2010). A phase III clinical trial (NCT02168842; www.clinicaltrials.gov) to study the neuroprotective potential of Isradipine in early PD patients is currently ongoing and scheduled for completion in 2019 (Table 2). While the contribution of Ca_v_1.3 channels to PD is undeniable, it is important to point out that Isradipine has, in fact, a higher affinity for Ca_v_1.2 channels (Koschak et al., 2001; Lipscombe et al., 2004; Olson et al., 2005; Xu and Lipscombe, 2001). Ca_v_1.2 channels are expressed throughout the brain and play important roles in regulating neurotransmitter release, predominantly at presynaptic terminals (Berger and Bartsch, 2014; Striessnig et al., 2006). Given that the exact roles of Ca_v_1.2 channels in PD have not been fully elucidated, this should be an important consideration when interpreting the results of these clinical trials.

**Table 2.**
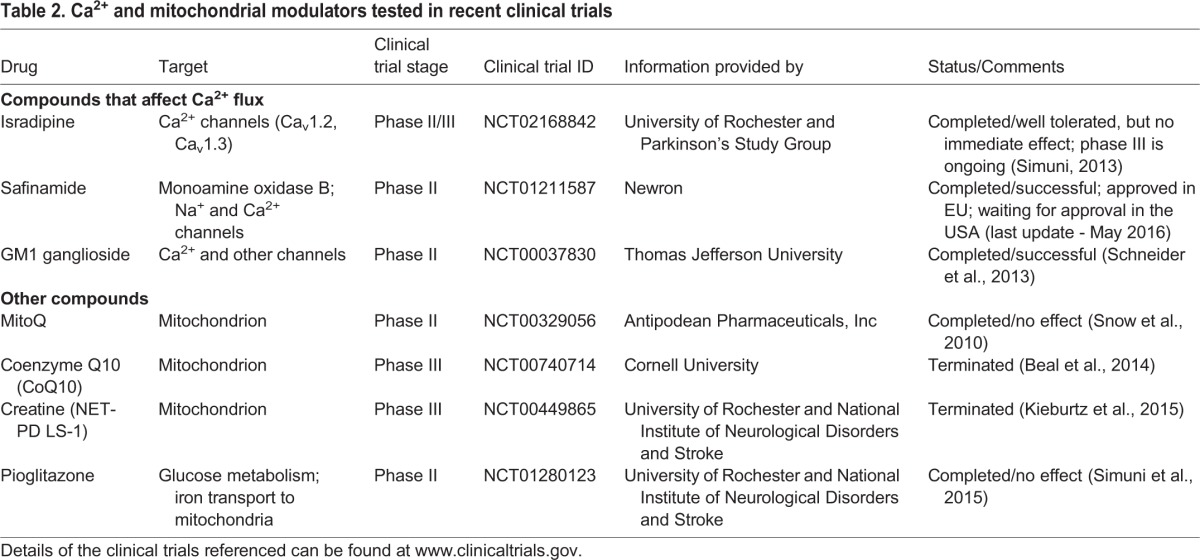
**Ca^2+^ and mitochondrial modulators tested in recent clinical trials**

Additional evidence also suggests a pathological role for α-synuclein in the increased influx of Ca^2+^ through the plasma membrane in PD. α-Synuclein can directly control the influx of Ca^2+^ through the plasma membrane by forming a specific type of oligomer, which can form Ca^2+^-permeable pores at the plasma membrane and induce cell death through Ca^2+^ (excitotoxicity) (Angelova et al., 2016; Danzer et al., 2007; Di Scala et al., 2016). Moreover, loss of function of Rab39B, a small GTPase that is involved in endosome trafficking and that is associated with early-onset PD, has recently been shown to alter the trafficking of an α-amino-3-hydroxy-5-methyl-4-isoxazole propionic acid receptor (AMPAR) subunit and to steer AMPAR toward forming a Ca^2+^-permeable channel (Table 1) (Lesage et al., 2015; Mignogna et al., 2015).

**Fig. 2.**
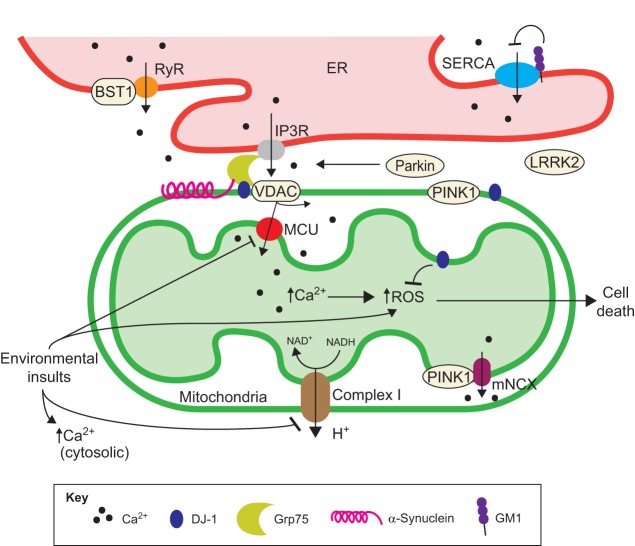
**Ca^2+^ signaling and homeostasis at ER-mitochondria contact sites.** A schematic of the mitochondria-associated membranes (MAMs) in the context of the PD-associated proteins (α-synuclein, PINK1, DJ-1 and BST1) involved in ER-mitochondria Ca^2+^ homeostasis. VDAC coupled with MCU mediates Ca^2+^ flow between the ER and mitochondria through its physical interaction with the IP3R via the Grp75 chaperone. ER Ca^2+^ homeostasis is also regulated by the RyR and SERCA pumps. BST1 activates RyR and depletes ER Ca^2+^, whereas GM1 inhibits SERCA-dependent ER Ca^2+^ uptake. PD-related environmental toxins (such as paraquat, MPTP and rotenone) lead to inhibition of MCU and Complex I, and to a concomitant increase in ROS and cytosolic Ca^2+^. Increased levels of mitochondrial Ca^2+^ can also lead to an increase in ROS and ultimately to cell death. Ca^2+^ is pumped out of mitochondria via Ca^2+^ exchange channels, such as the mitochondrial Na^+^/Ca^2+^ exchanger (mNCX), which is regulated by PINK1. DJ1 is a ROS scavenger that protects cells from ROS-induced cell death. DJ1, along with α-synuclein, interacts with Grp75 and promotes the formation of ER-mitochondria contact sites. Abbreviations: BST1, bone marrow stromal cell antigen-1; Complex 1, NADH coenzyme Q oxidoreductase; DJ1, protein deglycase; ER, endoplasmic reticulum; GM1, monosialotetrahexosylganglioside; Grp75, glucose-regulated protein 75; H^+^, hydrogen ion (protons); IP3R, inositol trisphosphate receptor; LRRK2, leucine-rich repeat kinase 2; MCU, mitochondrial Ca^2+^ uniporter; MPTP, 1-methyl-4-phenyl-1,2,3,6-tetrahydropyridine; NAD^+^, oxidized form of nicotinamide adenine dinucleotide; NADH, reduced form of nicotinamide adenine dinucleotide; Parkin, ligase encoded by the *PRKN* (*PARK2*) gene; PD, Parkinson’s disease; PINK1, PTEN-induced putative kinase 1; ROS, reactive oxygen species; RyR, Ryanodine receptor; SERCA, sarco/endoplasmic reticulum Ca^2+^-ATPase; VDAC, voltage-dependent anion channel type 1.

Finally, monosialotetrahexosylganglioside (GM1), a member of the sialic acid-containing glycosphingolipids group that is highly expressed at the plasma membrane of neural cells, appears to have an important role in neuronal Ca^2+^ homeostasis. GM1 can modulate several receptors and membrane channels, including Ca^2+^-ATPase (PMCA), Na^+^/Ca^2+^ exchanger (NCX), T-type Ca^2+^ channels at the plasma membrane, and sarco/endoplasmic reticulum Ca^2+^-ATPase (SERCA) pumps, to reduce excitotoxicity and oxidative stress (Hatzifilippou et al., 2008; Ledeen and Wu, 2015; Svennerholm et al., 1994). GM1 is also neuroprotective in rodent models of PD (Figs 1 and 2) (Schneider, 1998). In support of its protective role against PD pathogenesis, a completed phase II clinical trial in which PD patients were treated with GM1 (NCT00037830; www.clinicaltrials.gov) reported an overall improvement in the patients' motor symptoms and a delay in symptom progression during the two and a half-year trial period (Table 2) (Schneider et al., 2013, 2010).

## Intracellular Ca^2+^ stores and their deregulation in PD

Although the above evidence suggests that increased Ca^2+^ influx at the plasma membrane significantly contributes to the pathogenesis of PD, other findings implicate another form of Ca^2+^ deregulation in PD pathology. These findings report defects in the regulation of Ca^2+^ that comes from a cell's intracellular Ca^2+^ stores (Caraveo et al., 2014). The Ca^2+^ reservoir(s) responsible, as well as the mechanism behind this Ca^2+^ deregulation, have yet to be fully elucidated. Although the endoplasmic reticulum (ER), and to a lesser extent the mitochondria, are major intracellular Ca^2+^ stores, evidence suggests that other organelles, such as the lysosomes and Golgi, also act as important intracellular Ca^2+^ reservoirs (Kilpatrick et al., 2013; Patel and Docampo, 2010; Patel and Muallem, 2011). This is particularly relevant in the context of PD given that the malfunctioning of ER, mitochondria and, recently, lysosomes has been implicated in its etiology (Kilpatrick et al., 2016; Lloyd-Evans et al., 2008). Whether the deregulation of intracellular, store-derived Ca^2+^ plays a role in PD pathogenesis remains to be determined. One has to keep in mind that Ca^2+^-harboring organelles are not isolated static units but rather that they are highly dynamic and connected through a continuum of Ca^2+^ signaling. For example, the ER network is highly connected with many organelles through Ca^2+^-dependent pathways, including the plasma membrane, mitochondria, lysosomes and possibly other organelles (Bahar et al., 2016; Berridge et al., 2003; Bezprozvanny, 2010; Bootman, 2012; Calí et al., 2011; Carafoli, 2002; McBrayer and Nixon, 2013; Phillips and Voeltz, 2016; Rivero-Rios et al., 2014; Wojda et al., 2008). Connections also exist between lysosomes and peroxisomes, as well as between lysosomes and mitochondria. These interconnections are particularly relevant in the context of PD because it might support the argument for a ‘domino’ effect rather than an independent collection of defects, that is, if the ER is the first malfunctioning organelle, the other organelles that the ER is connected to, such as mitochondria and/or peroxisomes, will secondarily be affected. These organelles will, in turn, affect others that they are connected to and so on.

### Ca^2+^ storage in the ER

The ER is a major Ca^2+^ storage organelle in the cell and is responsible for protein biosynthesis and N-linked glycosylation. ER-derived Ca^2+^ plays crucial roles in cell signaling and also serves as a protein quality control system in the ER lumen. For example, a drop in ER luminal Ca^2+^ caused by misfolded proteins, such as α-synuclein, can lead to ER stress by halting protein translation and initiating the unfolded protein response (Celardo et al., 2016; Lindholm et al., 2006; Omura et al., 2013; Tsujii et al., 2015). Although this is a part of the normal physiological response to stress, a chronic ER system overload – which is observed in PD – can lead to cell death due to severe problems in cytosolic Ca^2+^ homeostasis, in protein biosynthesis, in Ca^2+^-mediated signaling pathways and in other organelle functions that are highly dependent on ER contacts (as discussed later in this review). Interestingly, some PD-associated genes encode proteins that are involved in depleting Ca^2+^ from ER stores. For example, the gene *BST1* (Bone marrow stromal cell antigen-1) is associated with sporadic PD in the European population (Table 1, Fig. 2) (Saad et al., 2011). BST1 is an adenosine diphosphate ribose (ADP) cyclase that can regulate Ca^2+^ release from the ER through the ryanodine receptors (RyR) by generation of cyclic ADP ribose (cADPR), a potent and universal Ca^2+^ mobilizer (Bruzzone et al., 2003; Nayak and De, 2007).

An additional link between ER Ca^2+^ homeostasis and PD is provided by phospholipase A2G6 (PLA2G6). Autosomal recessive mutations in *PLA2G6* lead to early-onset dystonia-parkinsonism (Tomiyama et al., 2011). PLA2G6 is a Ca^2+^-dependent phospholipase A2 that is associated with the plasma membrane (Table 1, Fig. 1). It normally interacts with the ER-Ca^2+^ sensor stromal interaction molecule 1 (STIM1) and promotes refilling of the intracellular Ca^2+^ stores via activation of Ca^2+^ channels at the plasma membrane, a process called store operated Ca^2+^ entry (SOCE) (Oslowski et al., 2013). PLA2G6 loss of function impairs SOCE, thereby decreasing the appropriate refilling of the ER with Ca^2+^. Disruption of SOCE also leads to autophagic dysfunction, progressive loss of DA neurons in SNc and age-dependent L-3,4-dihydroxyphenylalanine (L-DOPA)-sensitive motor dysfunction in animal models (Oslowski et al., 2013; Smani et al., 2004; Zhou et al., 2016). In support of a role for SOCE in the normal physiology of DA neurons in the SNc, overexpression of a dominant-negative form of the SOCE channel in the *Drosophila* brain, Orai1 (see Fig. 1), decreases expression of both tyrosine hydroxylase (TH) and the dopamine transporter (DAT) (Pathak et al., 2015). These data indicate that SOCE is important for maintaining the appropriate levels of dopamine in a normal brain and that alterations in this pathway might lead to PD-like pathology.

Defects in ER Ca^2+^ homeostasis can also have profound effects on other organelles through their physical connections. A good example of such interconnections is the ER-mitochondria contact sites, which form via mitochondria-associated membranes (MAM). As we discuss in the following section, these membranes are involved in several key processes, such as phospholipid and Ca^2+^ transfer, mitochondrial fission, mitophagy, the ER-stress response, and the regulation of apoptosis and inflammatory/antiviral responses (Vance, 2014).

### Ca^2+^ storage in the mitochondria

Mitochondria can temporally and spatially regulate cytosolic Ca^2+^ concentrations in distinct locations in a neuron. Aberrations in mitochondrial Ca^2+^ levels and in mitochondrial localization after organelle repositioning have been implicated in the pathogenesis of several neurodegenerative diseases, including PD (Fluegge et al., 2012; Rizzuto et al., 2012; Sheng and Cai, 2012). It is well established that mitochondrial Ca^2+^ overload can lead to oxidative stress – the increased production of reactive oxygen species (ROS) – and to changes in mitochondrial membrane permeability, both of which culminate in cell death (Krols et al., 2016; Lemasters et al., 2009; Marchi et al., 2014; McCormack and Denton, 1990). Indeed, defects in mitochondrial dynamics (fusion/fission and transport) and quality control are important contributors to PD pathology. Multiple PD-associated proteins [including α-synuclein, PINK1, DJ-1, Parkin and leucine-rich repeat kinase 2 (LRRK2)] are directly involved in regulating mitochondrial function, fusion/fission and oxidative stress (Table 1, Fig. 2), and are described in detail in many recent reviews (Bose and Beal, 2016; Calí et al., 2011, 2012a; Exner et al., 2012; Hu and Wang, 2016; Perier and Vila, 2012; Pickrell and Youle, 2015; Ryan et al., 2015). Importantly, treatment with Isradipine reduces mitochondrial oxidation and decreases the production of ROS in the SNc DA neuron in the *DJ-1* knockout mouse (Guzman et al., 2010). This finding strongly supports the argument that Ca^2+^ has a causal role in controlling ROS production, a key pathological feature of PD. Additionally, exposure of isolated mitochondria or cultured neuroblastoma cells to environmental insults such as MPTP and rotenone lead to a drop in mitochondrial Ca^2+^ influx and to a consequent increase in cytosolic Ca^2+^ (Frei and Richter, 1986; Sousa et al., 2003; Wang and Xu, 2005). Whether mitochondrial damage is locally generated and/or a consequence of the connections between organelles (such as the ER or peroxisomes) remains to be determined. Regardless, the high energy levels required to maintain Ca^2+^ homeostasis can explain why mitochondrial abnormalities could result in defective Ca^2+^ handling, as observed in PD. Given the importance of mitochondria in PD, four clinical trials (NCT00329056, NCT00740714, NCT00449865, NCT01280123; www.clinicaltrials.gov) have been conducted in the last 10 years that have aimed at improving mitochondrial health during the course of the disease (Table 2). However, after promising early stages, none of the drugs tested in these trials has proven to be effective at improving motor symptoms in PD patients (Beal et al., 2014; Kieburtz et al., 2015; Simuni et al., 2015; Snow et al., 2010). Nevertheless, mitochondrial health and mitochondria-associated proteins remain an attractive target for developing future therapeutics for PD.

As mentioned earlier, mitochondria Ca^2+^ levels are tightly controlled by the ER via MAMs. MAMs are enriched with the mitochondrial Ca^2+^ uniporter (MCU) complex in the inner mitochondrial membrane and with the inositol trisphosphate receptor (IP3R) on the ER. MCU and IP3R are coupled via the chaperone protein Grp75, which connects IP3R to the voltage-dependent anion channel type 1 (VDAC1) on the outer mitochondrial membrane (Fig. 2) (Krols et al., 2016; Rizzuto et al., 2009). These connections allow for Ca^2+^ exchange between ER and mitochondria, and tight regulation of mitochondrial luminal Ca^2+^ concentration. Mitochondrial luminal Ca^2+^ is essential for the Krebs cycle and for driving the electron transport chain through complexes III and V (Gellerich et al., 2013; Glancy and Balaban, 2012). Both biochemical processes are vital for maintaining the mitochondrial membrane potential and ATP levels. A cell needs sufficient energy to regulate Ca^2+^ owing to the high-energy demands of Ca^2+^ homeostasis. Mitochondria export Ca^2+^ via the H^+^/Ca^2+^ exchanger (mHCX) and the Na^+^/Ca^2+^ exchanger (mNCX), which are located on the inner mitochondrial membrane. Although the exact mechanism of action has yet to be established, two PD-associated proteins affect these mitochondrial Ca^2+^ import pathways: PINK1, by triggering the mNCX, and Parkin by stimulating VDAC1 (Table 1, Fig. 2) (Calí et al., 2013; Gandhi et al., 2009; Rizzuto et al., 2012). Moreover, α-synuclein and DJ-1 have both been shown to interact with MAM via the chaperone Grp75 (Jin et al., 2007). These interactions promote MAM assembly and function by controlling ER-mitochondria Ca^2+^ and lipid homeostasis (Table 1, Fig. 2) (Calí et al., 2012b; Guardia-Laguarta et al., 2014; Ottolini et al., 2013). These data suggest that disruption of MAMs might also be an important contributor to the pathogenesis of PD.

### Ca^2+^ storage in lysosomes and other acidic organelles

Lysosomes and autolysosomes are particularly important organelles for neuronal health given their long-lived nature and the high demand for constant nutrient turnover. Defects in autophagy and lysosomal function have both been observed in PD (Lynch-Day et al., 2012; Nixon, 2013; Xilouri et al., 2016). One of the strongest links between lysosomal function and PD is found with the enzyme β-glucocerebrosidase (GBA) (Table 1, Fig. 1). Autosomal recessive forms of the gene encoding this enzyme, *GBA*, cause the lysosomal storage disorder Gaucher's disease, which is characterized by the accumulation of glucosylceramide in hepatocytes. Individuals carrying a subset of these *GBA* mutations are 20 times more susceptible to developing PD (Beavan and Schapira, 2013; Dehay et al., 2013; Sidransky et al., 2009). This is likely to be due to the increased accumulation of α-synuclein aggregates in the lysosome due to the inability to degrade α-synuclein (Schapira et al., 2014). Interestingly, a defect in lysosomal trafficking caused by a PD-associated *GBA* mutation (L444P) was rescued in Gaucher patient-derived fibroblasts following treatment with the L-type Ca^2+^ channel blockers diltiazem or verapamil (Mu et al., 2008). Given that not all human carriers of *GBA* mutations develop PD, additional pathogenic mechanisms are likely to be at play.

Lysosomes and autosomes are especially important for the degradation of proteasome-insensitive protein aggregates, like those generated by α-synuclein; such degradation is dependent on lysosomal Ca^2+^ (Cuervo et al., 2004; Klionsky et al., 2010; Luzio et al., 2007, 2000). Lysosome-derived Ca^2+^ is thought to trigger Ca^2+^ release from the ER, possibly via lysosome-ER membrane contact sites (Kilpatrick et al., 2013; Phillips and Voeltz, 2016). Lysosomes mobilize their internal Ca^2+^ to signal in response to stimuli through a variety of channels, such as nicotinic acid adenine dinucleotide phosphate (NAADP)-dependent Ca^2+^ channels and members of the transient receptor potential channel superfamily, such as TPC (two-pore channels) and TRP (transient receptor potential channels) (Finbow and Harrison, 1997; Pitt et al., 2010). The Ca^2+^ content of these acidic compartments depends on the luminal pH, establishing a direct correlation between the efficiency of the proton pump V-ATPase and Ca^2+^ homeostasis (Christensen et al., 2002; Churchill et al., 2002; Guse and Lee, 2008; Lee et al., 2015). Such a link is evident in X-linked parkinsonism with spasticity (XPDS), which is associated with a mutation in *ATP6AP2*, which causes altered splicing of this prorenin receptor, a key regulator of V-ATPase function (Table 1, Fig. 1) (Jansen and Martens, 2012; Korvatska et al., 2013).

A mutation in *ATP13A2* links Ca^2+^ homeostasis and lysosomal function to autosomal recessive juvenile onset of PD (Table 1) (Di Fonzo et al., 2007; Ramirez et al., 2006; Ramonet et al., 2012; Santoro et al., 2011). ATP13A2 is a P5-type ATPase cation Mn^2+^ transporter that localizes to lysosomal membranes (Fig. 1). This protein protects against α-synuclein and Mn^2+^ toxicity in a yeast model of α-synuclein toxicity (Gitler et al., 2009). Mutant forms of ATP13A2 that are associated with PD mislocalize to the ER, causing defects in protein degradation and leading to parkinsonism that is levodopa-responsive (Di Fonzo et al., 2009; Matsui et al., 2013; Park et al., 2011; Schröder et al., 2007). Interestingly, silencing the expression of *ATP13A2* leads to a drop in cytosolic Ca^2+^ and to fragmented mitochondria in cortical neurons through an as yet unknown mechanism (Ramonet et al., 2012).

Three other proteins associated with autosomal-dominant forms of PD affect Ca^2+^ homeostasis both in the lysosome and in endosomal compartments. Two of these function in endosomal compartments: the vacuolar protein sorting 35 (VPS35), which is important for vesicular transport from the endosomes to the Golgi, and the vacuolar protein sorting 13 (VPS13C), which is important for vesicular transport from the Golgi to the endosomes. These proteins affect Ca^2+^ influx in the secretory pathway and protein recycling (Table 1) (Fokina et al., 2015; Mehta et al., 2016; Wang et al., 2016). The third is a lysosomal protein, LRRK2, that is linked through an unknown mechanism to the regulation of Ca^2+^ homeostasis in this organelle (Table 1) (Funayama et al., 2002; Gómez-Suaga et al., 2012; Gómez-Suaga and Hilfiker, 2012; Hockey et al., 2015).

Vesicles, another type of acidic organelle, are also highly affected in PD. Proper trafficking and priming of vesicles is crucial for synaptic function. Genes that are often mutated in PD encode proteins that have important functions in synaptic vesicle recycling, such as α-synuclein, LRRK2, TMEM230, SYNJ1, RIT2, SYT11, etc. (Table 1). So far, only a handful of these proteins are known to have a direct connection to Ca^2+^. α-Synuclein by itself can alter vesicle fusion by changing membrane curvature (Jensen et al., 2011; Nuscher et al., 2004), and it can also affect fusion by affecting the recruitment of several soluble NSF attachment (SNARE) proteins (Burre et al., 2010; Choi et al., 2013). Other evidence suggests that Ca^2+^ binding at the α-synuclein C-terminus can accelerate its aggregation, inhibiting its ability to bind to membranes and consequently promoting vesicle fusion (Table 1, Fig. 1) (Follett et al., 2013; Nath et al., 2011). Syntaxin 1B (STX1B) is an important member of the Ca^2+^-dependent proteins that mediate vesicle fusion at the plasma membrane (Südhof, 2013). A genome-wide association study identified the *STX1B* rs4889603 variant as a sporadic PD susceptibility locus in the Chinese population (Wang et al., 2015). Ca^2+^ binding to STX1B is necessary for the oligomerization of this protein and for the proper regulation of vesicle docking from the readily releasable pool at the synapse, although the underlying mechanism for this binding remains unknown (Table 1, Fig. 1) (Milovanovic et al., 2016; Mishima et al., 2014).

### Ca^2+^ storage in the Golgi

All lysosomal and secreted proteins are trafficked through the Golgi, the organelle responsible for the O-linked glycosylation of proteins and for the generation of endosomes for the secretory pathway. Although glycosylation enzymes inside the Golgi are highly dependent on internal Ca^2+^, the contribution of cytosolic Ca^2+^ to the Golgi has yet to be fully elucidated. Nevertheless, increases in cytosolic Ca^2+^ in neurons can lead to Golgi fragmentation, a reversible process mediated by CaMKII and/or CaMKIV (Thayer et al., 2013). Golgi fragmentation has been observed in cellular and animal models of PD, as well as in post-mortem brain samples from PD patients (Fujita et al., 2006; Gosavi et al., 2002; Lin et al., 2012; Rendón et al., 2013). Interestingly, two related Ca^2+^ channels, TRPV5 and TRPV6, can increase Ca^2+^ influx into the cytoplasm when glycosylated in *Xenopus laevis* oocytes (Jiang et al., 2008). Syntaxin 6 (STX6) inhibits TRPV channel glycosylation to allow their activation (Table 1). Although the role of STX6 in DA neurons is not known, the *STX6* rs1411478 variant is associated with progressive supranuclear palsy (PSP), a neurodegenerative disease that shares some characteristics with PD (Höglinger et al., 2011). Another connection between the Golgi and PD comes from the discovery that the yeast Ca^2+^/Mn^2+^ pump, PMR1 (homologous to the plasma membrane Ca^2+^-ATPase 1 in mammalian cells), is a modifier of cytosolic Ca^2+^ and α-synuclein toxicity in yeast (Büttner et al., 2013; Cooper et al., 2006). The role of Golgi as Ca^2+^ reservoirs in PD pathology remains unclear at present.

## Cytosolic Ca^2+^ signaling hubs and PD

Regardless of whether Ca^2+^ derives from the extracellular environment or from intracellular stores, what makes it such a powerful second messenger is its ability to affect protein conformation and ultimately protein function (Berridge et al., 2003; Clapham, 2007). An essential transducer of Ca^2+^ gradients into cellular responses is the highly evolutionary conserved protein calmodulin (CaM) (Fig. 1) (Chin and Means, 2000; Meador et al., 1992, 1993). CaM binds to Ca^2+^ via helix-loop-helix Ca^2+^-binding motifs called EF-hands. Ca^2+^ binding causes a large conformation change in CaM that exposes a hydrophobic surface capable of binding a diverse array of proteins (Table 1) (Gifford et al., 2007; Grabarek, 2011; Kawasaki et al., 1998; Lewit-Bentley and Réty, 2000). Binding of CaM to α-synuclein accelerates α-synuclein fibril formation *in vitro*, potentially contributing to PD pathogenesis (Lee et al., 2002; Martinez et al., 2003). Interestingly, some of the proteins implicated in PD are directly regulated by CaM, although the functional significance of these interactions in the context of PD has not yet been established. These include: RIT2 (Rin), a GTP-binding protein that is involved in DA neuronal function by regulating DAT trafficking and consequently extracellular dopamine concentrations (Table 1, Fig. 1) (Lee et al., 1996; Navaroli et al., 2011; Pankratz et al., 2012; Shi et al., 2005; Zhou et al., 2011); and DnaJ heat shock protein family member C13 (DNAJC13), which is involved in endocytosis and membrane trafficking through early endosomes (Table 1, Fig. 1) (Fujibayashi et al., 2008; Shen et al., 2005; Vilarino-Guell et al., 2014; Zhang et al., 2001).

Many CaM actions rely on activating and/or inhibiting downstream effectors that will, in turn, contribute to the pathogenesis of PD. One of the CaM-Ca^2+^ effectors is CaMKII, one of the most predominant protein kinases in the brain that is particularly important for synaptic function, learning and memory (Fig. 1) (Coultrap and Bayer, 2012; Hudmon and Schulman, 2002). Although the presynaptic role of CaMKII in PD is not fully understood, it has a role in both the initiation and prevention of dopamine release (Hoover et al., 2014; Michael et al., 2006; Wang, 2008). CaMKII also phosphorylates several microtubule-associated proteins, such as Tau, leading to defects in cytoskeleton dynamics and intracellular trafficking (Table 1, Fig. 1) (Baratier et al., 2006; Hashimoto et al., 2000; Johnson and Foley, 1993; Wang et al., 2007; Wei et al., 2015; Yamauchi and Fujisawa, 1983, 1984). The hyperphosphorylation of Tau contributes to its aggregation and thus to the consequent formation of neurofibrillary tangles, the pathological hallmark of tauopathies, such as Alzheimer’s disease (Wang et al., 2013a). Moreover, CaMKII and phosphorylated Tau are found in Lewy bodies, suggesting an important role for these proteins in the etiology of PD (Iwatsubo et al., 1991; McKee et al., 1990; Moussaud et al., 2014). CaMKII also phosphorylates TH, the rate-limiting enzyme in the biosynthesis of catecholamines, such as dopamine, noradrenaline and adrenaline, and increases dopamine synthesis (Fitzpatrick, 1999; Albert et al., 1984; Lehmann et al., 2006). Abnormal increases in cytosolic dopamine are reportedly neurotoxic in cultured rat midbrain DA neurons. Importantly, reducing cytosolic Ca^2+^ significantly decreases cytosolic dopamine and prevents toxicity in DA neurons in the rat SNc (Mosharov et al., 2009).

Another essential transducer of Ca^2+^ signaling is the highly conserved Ca^2+^-CaM-dependent serine/threonine phosphatase calcineurin. Calcineurin is an essential enzyme, which in the adult brain plays a key role in neurite extension, synaptic plasticity, memory and learning (Zeng et al., 2001), and is implicated as a key mediator of α-synuclein toxicity (Table 1) (Caraveo et al., 2014; Martin et al., 2012). Most importantly, our group has found that persistent and excessively high levels of calcineurin activity caused by α-synuclein drive dephosphorylation of target proteins, such as the nuclear factor of activated T cells (NFAT), setting up a program that leads to cell death (Fig. 1) (Caraveo et al., 2014; Luo et al., 2014). However, low levels of calcineurin activity, achieved with low doses of the calcineurin specific inhibitor (FK506) or by genetic means, lead to the dephosphorylation of a distinct subset of proteins, such as the target of rapamycin complex 2 (TORC2), which protects cells from the toxic effects of α-synuclein (Fig. 1). The complete inhibition of calcineurin with high doses of FK506 or deletion of the calcineurin gene eliminates its ability to dephosphorylate any target proteins, which also leads to cell death (Caraveo et al., 2014). We named this the ‘Goldilocks’ effect, where too much or no activity leads to cell death, but an intermediate level of activity is neuroprotective.

In addition to NFAT and TORC2, there are other calcineurin substrates implicated in PD. These include the transcription factor cAMP-responsive element binding (CREB), which has important roles in synaptic plasticity and long-term memory formation, and which is activated by phosphorylation and repressed in a calcineurin-dependent manner (Marambaud et al., 2009; Sakamoto et al., 2011). As a surrogate for high calcineurin activity, CREB has been found to be repressed in both primary mouse DA neurons treated with the neurotoxin 6-hydroxydopamine (6-OHDA) and in human PD brain samples (Chalovich et al., 2006; Sakamoto et al., 2011). Another calcineurin substrate is Synaptojanin 1 (SYNJ1), a lipid phosphatase that, when dephosphorylated by calcineurin, enhances clathrin-mediated endocytosis (Table 1, Fig. 1). Mutations in *SYNJ1* that affect its phosphatase function are associated with early-onset progressive parkinsonism with generalized seizures (EOP) (Krebs et al., 2013; Lee et al., 2004). EIF2AK3, also known as protein kinase RNA-like endoplasmic reticulum kinase (PERK), couples ER stress to translation inhibition during protein misfolding (Table 1, Fig. 1) (Mercado et al., 2015). *EIF2AK3* is a risk gene associated with progressive supranuclear palsy (PSP) (Höglinger et al., 2011). Although the effect of the single nucleotide polymorphism (SNP) associated with PSP is unknown, it is noteworthy that the interaction of calcineurin with PERK promotes PERK auto-phosphorylation, leading to translation inhibition (Bollo et al., 2010). In support of the role of PERK in the pathology of PD, phosphorylated PERK is found in SNc DA neurons from deceased PD patients, as well as in Lewy bodies (Hoozemans et al., 2007). In addition, another substrate of calcineurin, calnexin (CNX, an ER-resident chaperone), when dephosphorylated, releases the block caused by SERCA pumps and restores Ca^2+^ homeostasis in the ER (Bollo et al., 2010; Wang et al., 2013b). Although the ‘Goldilocks’ property of calcineurin has been demonstrated on just a handful of substrates, many other targets are likely to be involved with α-synuclein toxicity that remain to be discovered.

Another interesting Ca^2+^-dependent group of enzymes implicated in PD are calpains. These cytosolic cysteine proteases are involved in the regulation of synaptic plasticity and long-term potentiation. Acute calpain activation is beneficial to neurons, whereas chronic activation induced by sustained cytosolic Ca^2+^ can lead to cell death. In support of a role for Ca^2+^ deregulation in PD, over-activated calpains have been detected in postmortem PD brains (Crocker et al., 2003; Mouatt-Prigent et al., 2000; Samantaray et al., 2008). Moreover, bioinformatic analysis has revealed two single SNPs in the gene encoding the only endogenous inhibitor of calpain, the calpastatin gene (*CAST*), which might predispose Caucasian individuals with European ancestry to idiopathic PD (Allen and Satten, 2009, 2010; Dauer and Przedborski, 2003). Some studies suggest that calpains have a protective role in PD through promotion of α-synuclein degradation via the modulation of the E3 ubiquitin ligase Parkin (Kim et al., 2003), whereas others point to their having two possible toxic roles. First, calpains can promote α-synuclein aggregation *in vitro* and *in vivo* by cleaving the α-synuclein C-terminal domain (Table 1, Fig. 1) (Diepenbroek et al., 2014; Dufty et al., 2007; Nuber and Selkoe, 2016; Xu et al., 2015). Second, calpains can cleave p35 (CDK5R1). The p35 activates Cdc5, a cyclin-dependent kinase that has a key role in neuronal development (Ko et al., 2001; Ohshima et al., 1996), axonal transport (Julien and Mushynski, 1998), synaptic activity (Rosales et al., 2000) and dopamine signaling (Chergui et al., 2004; Nishi et al., 2002). In MPTP-treated animals and in α-synuclein cell model systems, the activation of calpain leads to p35 being cleaved into its pathological form, p25, which results in the mislocalization and hyperactivation of Cdk5, and in DA neuronal loss in the mouse SNc (Czapski et al., 2013; Smith et al., 2006). p25 and overactive Cdk5 are detected in PD animal models (Qu et al., 2007; Smith et al., 2003) and in Lewy bodies from postmortem PD brains (Alvira et al., 2008; Takahashi et al., 2000). Importantly, the inhibition of calpains is effective at reducing overactive Cdk5 and p25, and is protective against toxicity in animal models of PD (Chagniel et al., 2012). In addition, the peptide TFP5, which is derived from p35, is reported to be neuroprotective in the MPTP-treated rat cortical neurons and a mouse model of PD (Binukumar and Pant, 2016; Binukumar et al., 2015; Zhang et al., 2012).

## Cytosolic Ca^2+^ buffering and PD

As we mentioned earlier, an important contributor to the vulnerability of SNc DA neurons in PD is their inability to buffer Ca^2+^, caused by decreased expression of Ca^2+^-buffering proteins such as calbindins and parvalbumin. Calbindins, which include calbindin-D28k (encoded by *CALB1*) and calbindin-D9k (encoded by *S100G*), are vitamin D-dependent Ca^2+^-binding proteins. Calbindin-D9k is mostly known for buffering Ca^2+^ in erythrocytes, whereas calbindin-D28k buffers Ca^2+^ in the central nervous system where it participates in the blockade of multiple pro-apoptotic pathways (Baimbridge et al., 1992). Overexpression of calbindin-D28k in the midbrain ventral tegmental DA neurons is associated with reduced cell death in human PD samples and in mouse models of the disease, compared to the calbidin-D28k-negative more vulnerable SNc DA population (Damier et al., 1999; Lavoie and Parent, 1991). Reduced expression of Ca^2+^-buffering proteins, as well as a chronic increase in intracellular Ca^2+^ in aging SNc DA neurons, are likely to be mechanisms that contribute to the mitochondrial and ER stress observed in PD. Mice overexpressing calbindin-D28k are resistant to the toxic effects of MPTP and to α-synuclein aggregation (Rcom-H'cheo-Gauthier et al., 2016; Yuan et al., 2013), establishing a causal link between buffering Ca^2+^ and protection against cell death. Interestingly, calbindin-D28k is also a reported risk factor for sporadic forms of PD in a Japanese population (Mizuta et al., 2008; Soto-Ortolaza et al., 2010).

Parvalbumin (PA) is another Ca^2+^-binding protein that is selectively expressed in a class of GABAergic interneurons of the dorsolateral prefrontal cortex (Benes and Berretta, 2001; Kretsinger and Nockolds, 1973), a region also affected in PD patients (Kikuchi et al., 2001). Altered PA levels are likely to contribute to the altered cortical excitability and oscillatory activity previously documented in PD (Lanoue et al., 2013). Moreover, loss of PA-positive neurons is reported in animal models of PD and in human PD brain samples (Fernández-Suárez et al., 2012). This suggests that decreased PA expression is associated with defects in Ca^2+^ buffering and cell death.

## Discussion

Given the universal nature of Ca^2+^ signaling in biology, its involvement in the etiology of PD and other neurodegenerative disorders (Box 1) might not come as a surprise. Although it is now increasingly recognized that gradual Ca^2+^ dysregulation might be a key contributor for aging, what distinguishes its contribution to PD from that to normal aging and/or other neurological diseases is that many of the genes that give rise to PD have a known causal role in Ca^2+^ homeostasis. As we have described in this review, compelling evidence implicates the deregulation of Ca^2+^ flux both from the plasma membrane (through mechanisms involving Ca_v_1.3, α-synuclein pore formation, etc.) and from intracellular stores (through other mechanisms involving α-synuclein and GBA, among others). As such, understanding the mechanisms by which Ca^2+^ signaling contributes to the progression of PD is vitally important for developing effective therapies to treat this disease.

Most of the organelles affected in PD are major Ca^2+^ reservoirs. This suggests that Ca^2+^ could be a key player in coordinating complex organelle networks to ultimately achieve metabolic interactions, intracellular signaling, cellular maintenance and regulation of cell survival. Although neuronal cell culture models, *in vivo* rodent models and midbrain DA neurons derived from patient induced pluripotent stem cells (iPSCs) are vitally important tools for understanding the mechanisms underlying the pathology of PD, a significant investment of time and money is needed to make the most of these tools. Time is an issue when phenotypes are age dependent, as is the case for PD, and when the lifespan of rodents and/or primates is years. Even for iPSC-derived neurons, the time it takes in cell culture for any meaningful phenotype to appear can be up to a year. This, added to the high cost of performing mammalian-based experiments, makes these systems less than amenable for exploratory mechanistic research, despite the fact that they provide an essential validation tool for translation into the clinic. The use of model organisms such as yeast, flies and worms, can effectively circumvent these roadblocks. Indeed, these models have proven to be invaluable tools for uncovering conserved disease and cell biological processes that are affected in PD, which range from lipid biology, vesicular trafficking and function, lysosomal and peroxisomal function, autophagy, apoptosis, cell cycle, mitochondria and oxidative stress, Ca^2+^ signaling, ion channels and transporters, and the protein folding, quality control and degradation pathways. Model organisms can provide an excellent means to understand the mechanisms of Ca^2+^ deregulation in PD and could also shed light on how organelle networks operate to achieve cellular plasticity by using Ca^2+^ as a messenger, ultimately leading to novel therapeutic alternatives for combating PD.
